# Advancing In Vivo Molecular Bioimaging With Optimal Frequency Offset Selection and Deep Learning Reconstruction for CEST MRI

**DOI:** 10.1109/access.2025.3571638

**Published:** 2025-05-19

**Authors:** ADARSHA BHATTARAI, CHATHUMI SAMARAWEERA, MARIANO UBERTI, ADITYA N. BADE, YUTONG LIU, DONGMING PENG

**Affiliations:** 1 Department of Electrical and Computer Engineering, University of Nebraska–Lincoln, Omaha, NE 68182, USA; 2 Department of Radiology, University of Nebraska Medical Center, Omaha, NE 68198, USA; 3 Department of Pharmacology and Experimental Neuroscience, University of Nebraska Medical Center, Omaha, NE 68198, USA

**Keywords:** Biomedical signal processing, computer vision, image reconstruction, machine learning, artificial intelligence, magnetic resonance imaging, biomedical imaging, molecular imaging

## Abstract

Chemical exchange saturation transfer (CEST) magnetic resonance imaging (MRI) is an emerging non-invasive molecular imaging technique offering significant potential for biomedical research and clinical applications. However, CEST MRI data acquisition requires prolonged scanning times, as data need to be collected at multiple frequency offsets to capture necessary information for accurate analysis of biological compounds. Faster CEST MRI will improve molecular imaging, advancing biomedical pre-clinical research studies and clinical applications. Thus, herein, we accelerate CEST MRI data acquisition using a two-step approach. Firstly, we use an optimization algorithm to identify a set of optimal sparse frequency offsets for data collection. Secondly, we apply a deep learning algorithm to reconstruct the high-resolution CEST MRI Z-spectra from the low-resolution Z-spectra. CEST MRI data acquired on adult mice brains (n = 19) were utilized. The optimization technique efficiently selected down to 10% of the total frequency offset points, and the deep learning algorithm accurately reconstructed dense Z-spectra. The performance metrics, root mean square errors (RMSE), mean absolute error (MAE), and Pearson’s correlation were calculated for various Z-spectra reconstructions. The minimum, maximum, and average RMSE values achieved when the lowest 10% of frequency offsets were used were 0.0065, 0.0133, and 0.0094, respectively. The proposed CEST MRI approach, involving optimal frequency offset selection followed by deep learning reconstruction, achieves an acceleration by 10 times while maintaining high-quality data. This approach expands the applications of CEST MRI, potentially advancing *in vivo* molecular bioimaging for both basic science and clinical research.

## I. INTRODUCTION

Chemical exchange saturation transfer (CEST) based magnetic resonance imaging (MRI) is a promising non-invasive and quantitative molecular imaging technique [1], [2], [3], [4], [5], [6]. CEST MRI is employed in both pre-clinical and clinical applications, particularly for studying metabolic processes, detecting lesions, and assessing microenvironment properties such as pH and temperature [7], [8]. This imaging technique offers a powerful method to detect diluted molecules indirectly by capturing their saturation transfer to the large water pool at distinct frequency offsets [13]. CEST MRI can capture compounds that include protons showing an appropriate exchange rate with the bulk water [2]. These compounds, such as glucose, amino acids, or metabolites, can be naturally produced within the body or introduced externally, such as contrast agents or drugs. Furthermore, this imaging technique provides high specificity and excellent spatial resolution for detecting target molecules.

A major limitation of CEST MRI is the long scan time [14], [15], [16], [17], [18]. This challenge occurs because MRI signals need to be acquired at multiple saturation frequency offsets. The CEST acquisition time increases proportionally with the number of frequency offsets required to generate CEST contrast maps [13]. Typically, an accurate multipool evaluation of CEST data requires a densely sampled Z-spectrum over a broad frequency range, resulting in a long scanning time. Therefore, reducing the scan time by efficiently downsampling the frequency offsets and reconstructing the Z-spectrum with deep learning or other advanced techniques is highly desirable. Addressing this challenge is essential for the advancement of CEST MRI applications in biomedical investigations and clinical applications.

To minimize the acquisition time, various state-of-the-art deep learning methods have been proposed that reduce the number of k-space lines sampled in the phase encoding (PE) direction (*N*_*PE*_) [9], [10], [11], [12]. Combining such approaches that reduce *N*_*PE*_ and our approach to optimize the number of frequency offsets (*N*_*offs*_), there may be a potential to reduce the CEST MRI scan time multiplicatively. This is because both *N*_*PE*_ and *N*_*offs*_ are directly proportional to the acquisition time, given that the repetition time and the echo train length are constant [12].

Furthermore, a variety of deep learning methods have been suggested to speed up CEST data acquisition [13], [14], [17], [19]. These methods reconstruct detailed Z-spectra or contrast maps using limited frequency offsets. However, the selection of optimal frequency offsets remains uncertain. In this study, we tested different sets of frequency data to identify the optimal frequency offsets for the reconstruction of Z-spectra.

In our study, we propose a two-phase approach to address the limitation of the long scan time required for CEST MRI. In the first phase, we employ the genetic algorithm (GA) as the optimization algorithm. This approach provides a logical framework to determine the most suitable frequency offset points from the dense Z-spectra for data acquisition. In our study, we deal with a relatively smaller search space size of 101 offsets, which is different from other typical complex optimization problems, which may reach to search space size of millions. Therefore, we sought an optimization algorithm that would consider both diverse combinations of offsets from a small search space and produce high accuracy. GA was chosen and applied to select the optimal frequency offsets down to 30, 20, and 10 offsets from a total of 101 frequency offsets. Although GA has higher computational complexity and slower convergence speed than other algorithms, such as simulated annealing (SA), it was used because it considers diverse combinations of optimal solutions and is better at converging to the global optima and avoiding local optima. In contrast, SA may perform better when the search space is large, such as the size of millions. Moreover, past studies on GA and SA show that GA exhibits lower error when the learning size is small [20].

The optimal frequency offsets were selected to minimize errors in reconstructing dense Z-spectra. In the second phase, we utilize supervised deep learning algorithms, including autoencoder and U-Net models, to reconstruct CEST MRI from the sparse data. Through this framework, we reconstruct high-resolution Z-spectra from low-resolution Z-spectra. The proposed framework was tested on *in vivo* mouse brain CEST MRI data. Both the optimization algorithm and the deep learning models were evaluated for their accuracy in reconstructing Z-spectra. This approach develops a high-performance optimization and AI-based CEST acquisition scheme capable of reconstructing CEST MRI from limited data.

## II. RELATED WORKS

Chemical exchange saturation transfer magnetic resonance imaging (CEST-MRI) has emerged as a powerful tool, offering a unique insight into the complex biochemical processes [21], [22], [23]. Recently, CEST MRI-related studies have been conducted to investigate the functionalities of organs and tissues, as well as their significance in advancing our understanding of health and diseases [24], [25], [26], [27]. In these studies, special attention was given to reducing the scan time of the CEST MRI by incorporating the emerging AI technology [9], [10], [11], [12], [13], [14], [15], [16], [17], [18], [19].

Xu et al. have proposed model model-based deep neural network, CEST-VN, that takes undersampled k-space data as input and outputs CEST source images [9]. In addition, they proposed a specialized loss function specifically for CEST images to quantify error. Liu et al. have developed an unsupervised deep learning algorithm with enhanced hash encoding [10]. They presented high acceleration factors for CEST imaging compared to the current state-of-the-art deep learning method, with experiments performed on various datasets, including a phantom of fresh eggs, liver of mice, and a human brain dataset. Yang et al. have presented a deep learning reconstruction network with a multiple radial k-space sampling strategy for CEST MRI [11]. The input to the network model was three undersampled k-space data at 3 consecutive offsets, and the output was the reconstructed image representing the central offset. Prabakaran et al. have conducted a study on a deep learning based super-resolution approach to reconstruct high-resolution CEST images, which exhibited improved spatial resolution in terms of PSNR and SSIM performance metrics [12].

The acceleration factor for these studies ranged from 2 to 37.5 [9], [10], [11], [12]. This indicated that the researchers could reconstruct the CEST images from k-space data undersampled by factors ranging from 2 to 37.5. The fundamental difference between these acceleration approaches and our approach is that we focus on optimizing the number of frequency offsets (*N*_*offs*_) to accelerate CEST imaging. Furthermore, these approaches may work collaboratively to contribute to the common objective of reducing the acquisition time.

Cheema et al. have introduced a method to reduce CEST acquisition time by utilizing down-sampled Z-spectrum with deep learning to construct CEST maps [13]. In this method, the optimal frequency offsets were detected by utilizing the “Fisher offsets” based on parameters of Lorentzian functions for multi-pool CEST contrast fitting. Then, a U-Net was trained with down-sampled CEST images collected from 18 volunteers. The conventional multi-pool models produced unsatisfactory results (structural similarity index [SSIM] < 0.2, peak SNR < 20, and Pearson *r* < 0.1), whereas the proposed U-Net fitting method successfully processed the down-sampled images to quantify the CEST data [13]. Although Cheema et al.’s work has contributed to reducing the CEST acquisition time by utilizing Fisher’s Offsets to select the optimal frequencies, the criterion for the selection of optimal frequency offset points was not fully exploited. For instance, it is unknown if the method can select less than 15 out of 53 (28.3%) Z-spectrum offsets. In our research work, we reduce the CEST acquisition time by selecting up to 10% of the total frequency offset points. These efforts more efficiently reduce CEST acquisition time compared to the method proposed by Cheema et al.

Chen et al. have conducted a deep learning-based study to reduce the scan time while preserving the accuracy of multi-pool CEST MRI of patients with Parkinson’s disease [14]. An updated 1-D, U-Net (termed as Z-spectral compressed sensing (CS)) was utilized to reconstruct the dense Z-spectra with sparse frequencies. The results obtained from simulations and the *in vivo* rat brain testing proved the validity and high precision of the prediction of Parkinson’s disease while maintaining the scan time in an acceptable range (can be reduced up to 33%) compared to classical methods [14].

Liu et al. have developed a technique to expedite the Z-spectral acquisition process by incorporating deep learning-driven reconstruction and uniquely designed k-space sampling trend along with a frequency-offset-dependent (FOD) function [15]. To improve spatial and frequency domains individually, a convolutional neural network (CNN) was upgraded with a partially separable (PS) function. The integrated FOD sampling and PS network achieved the best reconstruction function, outperforming other classical sampling techniques in both retrospective and prospective tests [15].

Yang et al. have introduced a method to overcome the constraint of extended saturation time to approach the steady state of the CEST effect by employing a deep learning oriented quasi-steady-state (QUASS) estimation from non-steady-state CEST [16]. They have designed a hybrid structure of Long short-term memory (LSTM) - Attention network to enhance the prediction accuracy. The proposed framework has outrun QUASS CEST prediction compared to other deep-learning techniques such as multilayer perceptron, recurrent neural network, LSTM, gated recurrent unit, and BiLSTM [16].

Each of the researchers mentioned above has contributed to expediting the Z-spectral acquisition process by employing deep learning-based reconstruction techniques [14], [15], [16]. Although they have utilized sparse frequencies to reconstruct Z-spectra, they have not taken a systematic approach to choose the best set of optimal frequency points. In our study, this crucial aspect was addressed by utilizing an optimization algorithm to identify the optimal sparse frequency offsets from dense Z-spectra, enabling the reconstruction of high-resolution CEST MRI, which would outperform the previously proposed methods by Liu et al., Chen et al., or Yang et al. [14], [15], [16].

Perlman et al. have developed a technique for fast and quantitative CEST MR fingerprinting recreation and data acquisition by employing an automated machine learning-based method termed AutoCEST [18]. The AutoCEST framework was evaluated by using different chemical exchange models to acquire data on *in vivo* mouse brains. The evaluation results confirmed the ability to automatically create enhanced CEST/MT data acquisition and rapid reconstruction into quantitative interchange parameter maps [18].

Xiao et al. have presented a novel approach to reconstruct dense Z-spectra to reduce the scan time from the experimental images at sparse frequency offsets through a new sequence-to-sequence (seq2seq) model [19]. Experimental results proved the better efficiency and accuracy of the new seq2seq network in reconstructing the dense CEST images compared to the conventional seq2seq network [19].

The discussed literature confirms the unique and potential research angle on reducing the CEST MRI data acquisition time through the incorporation of AI technology. Each study offers a distinct perspective on the issue of lengthy scan times associated with CEST MRI, which helps to provide a broader understanding of this research problem. It is worth mentioning that these studies have made commendable efforts to reduce CEST MRI acquisition time and have made valuable contributions to the field of brain health and diseases.

Although the reviewed literature has made a remarkable contribution to accelerate CEST MRI data acquisition, very few have focused specifically on the selection of optimal frequency offset points. Through our proposed study, the challenge of prolonged scan times for CEST MRI data acquisition is addressed in two phases. Initially, the optimal set of sparse frequencies is acquired by employing an optimization algorithm, the genetic algorithm. In the second phase, a high-resolution Z-spectra is reconstructed with the support of deep learning, especially by utilizing the autoencoder and U-Net models. Therefore, the proposed approach to address this challenge is novel and efficient compared to the reviewed literature, and it will make an impact in advancing molecular imaging, benefiting biomedical studies, and clinical applications.

## III. METHODS

The acquisition time for CEST MRI (*T*_*A*_) as mentioned by Prabakaran et al. [12] is shown in Equation 1, where *TR* is the repetition time, *ETL* is the echo train length and *N*_*PE*_ is the number of k-space lines sampled in the phase encoding direction.


(1) $$T_{A}=\frac{TR\cdot N_{PE}}{ETL}\cdot N_{\text{offs}}$$

Our approach aims to optimize the frequency offsets (*N*_*offs*_) to reduce the acquisition time (*T*_*A*_) described in the following subsections.

### A. DATASETS

#### 1) DATA ACQUISITION

The study involved *in vivo* mice dataset (n = 19). In the study, C57BL/6 male mice aged 6 months were used. Mice brains were scanned on a 7 T Bruker PharmaScan system. Before scanning, the mice were anesthetized with isoflurane carried by oxygen, and their breathing was monitored throughout the experiment. To provide anatomical reference, each scan session started with T2-weighted imaging. To optimize *B*_0_ uniformity, Bruker’s MAPSHIM protocol was run at the beginning of the experiment to homogenize the magnetic field in the entire brain region. CEST data were acquired on two brain slices using a RARE sequence and a continuous RF saturation pulse with power = 2 μT, and length = 2 s. The frequency range was set from −5 to 5 ppm with a step = 0.2 ppm. The total number of frequency offsets was 51.

#### 2) MOTION, B0 FIELD DRIFT AND WASSR ESTIMATION

Long acquisitions are susceptible to motion artifacts. Our method substantially reduces scan time, making it more robust than traditional CEST MRI. To mitigate *B*_0_ field drift, we employ localized shimming, which effectively improves field homogeneity. In our study, we used Bruker’s MAPSHIM method for whole-brain shimming in mice. For *B*_0_ correction, we acquire high-resolution WASSR scans (5 minutes) before CEST MRI to establish an accurate *B*_0_ field map. To further ensure correction accuracy, WASSR results are cross-validated using Lorentzian fitting of the direct water saturation signal from the CEST scan. This dual approach enhances reliability in compensating for *B*_0_ inhomogeneities.

#### 3) PRE-PROCESSING

After the data acquisition from the MRI machine, *B*_0_ correction was carried out using WASSR [28] and linearly interpolated to 101 frequency offsets. Image segmentation was performed to remove non-brain tissue from the acquired 4D CEST MRI images of the mouse brain. The *B*_0_ correction and brain segmentation were performed using MATLAB and Medical Image Processing, Analysis, and Visualization (MIPAV) tools, respectively. After these operations, the 4D *in vivo* dataset for a single mouse subject is represented in Equation 2 as $M_{4D_{0}}$. *α* and *β* represent x and y coordinates of the pixel location in an acquired brain slice *γ* at frequency offset *φ*.

$$M_{4D_{0}}(\alpha ,\beta ,\gamma ,\phi ),$$

where *α, β* ∈ {x and y coordinates of the pixel},

(2) $$\gamma \in \left\{1,2\right\},\;\phi \in \left\{1,\ldots ,101\right\}.$$

In addition, the intensity of a pixel at location (*α, β*) in a brain slice *γ*, acquired at frequency offset *φ*, is represented by the function *P*(*α, β, γ, φ*).

Dataset A. For the mice dataset (n = 19), 38 dense Z-spectra were obtained by averaging all pixel values across the entire slice for each frequency offset. Later, the Z-spectra dataset was augmented using two methods.

Dataset A’. The first method added additive white Gaussian noise, with a mean of 0 and a standard deviation of 0.01, to each Z-spectra value in the first 30 Z-spectra of Dataset A, which consists of a total of 38 Z-spectra. The remaining 8 Z-spectra were separated without augmentation for testing. The dataset was augmented 100 times. The dataset consisted of 3000 total rows of Z-spectra values.

Dataset B. In the second method, the dataset was augmented using sub-samples of pixels across the frequency offsets for each brain slice. The range was set as [800:50:1550] as the number of pixels in the sub-samples. The sub-samples started with 800 pixels and increased up to 1550 pixels in increments of 50. A total of 16 sub-samples allowed to augment the dataset from 38 to 608 sets of Z-spectra values: (38 × 16) = 608. This dataset was divided into training and testing sets, containing 480 and 128 Z-spectra, respectively.

We obtained three different sets of datasets using these methods. We trained separate deep-learning reconstruction models using each dataset. These diverse datasets aimed to study the adaptation of deep learning models to diverse Z-spectra values. This was also an effort to enhance the generalizability of the deep learning model for reconstructing diverse Z-spectra.

#### 4) EVALUATION

The optimization algorithm and the supervised deep learning methods were evaluated on mouse datasets. The optimization algorithm was evaluated based on the accuracy of reconstructed Z-spectra using the optimal frequency offset points selected by the optimization algorithm. The genetic algorithm is a promising optimization technique for the study [29]. Likewise, the deep learning model was tested based on the reconstruction accuracy of Z-spectra. The sparse Z-spectra were used as input to the model, and the dense Z-spectra were generated as output. The root mean square errors (RMSE), mean absolute error (MAE), and Pearson’s correlation were used as the measurement metrics.

### B. ARCHITECTURE

**FIGURE 1.**
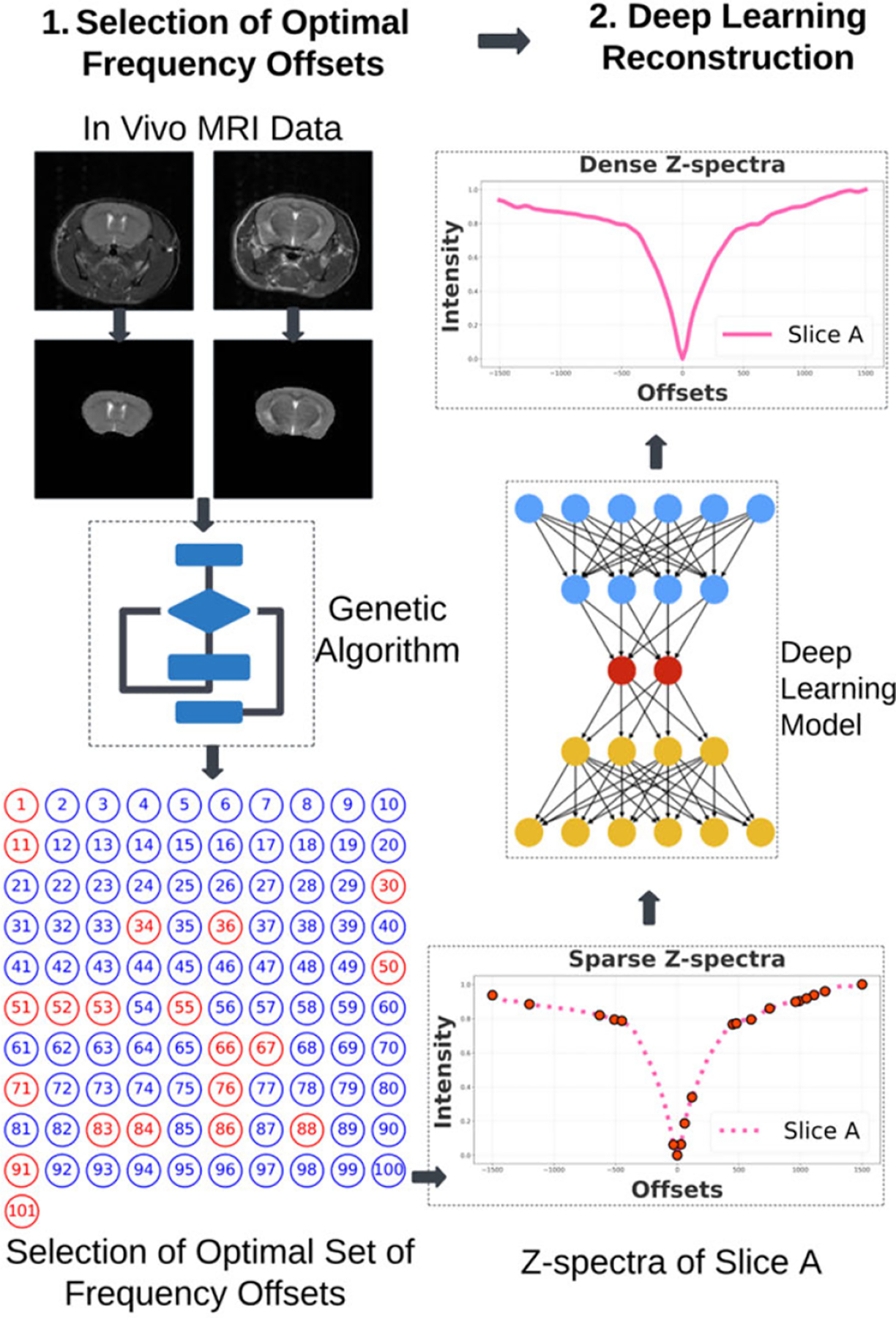
Proposed methodology shows (1) the optimization of frequency offset points followed by (2) deep learning-based reconstruction of high-resolution MRI data. In method (1), the genetic algorithm optimization selects the optimal frequency offsets based on the best fitness score, corresponding to accurate Z-spectra reconstruction. In method (2), the dense Z-spectra are reconstructed using the optimal frequency offsets.

The system architecture consists of two main steps as shown in Figure 1. First, the selection of optimal frequency offset points, and second, deep learning reconstruction. The genetic algorithm was used as the optimization algorithm, which selects the optimal frequency offset points. In our experiment, each dense Z-spectra was of dimension 1 × 101 and was inputted to the genetic algorithm to select *p* percent of the total frequency offset points. Consequently, a sparse Z-spectrum was obtained based on the optimal frequency offset selection. Later, the deep learning models based on autoencoder and U-Net architectures were used to reconstruct the dense Z-spectra. The autoencoder model architecture consisted of an upsampler and downsampler, whereas U-Net consisted of an additional layer, a skip connection. Initially, the autoencoder model was used to train with fewer datasets compared to the larger dataset used to train the U-Net model. The U-Net model was more complex in terms of architecture. However, both models were tested with various datasets, and their performances were evaluated.

### C. IMPLEMENTATION OF THE METHOD

#### 1) SPARSE FREQUENCY OFFSETS OPTIMIZATION

The optimal sparse frequency offsets were obtained using the genetic algorithm, which selects the best set of sparse frequency offsets that result in the minimum error while reconstructing the dense Z-spectra. The genetic algorithm took the number of sparse frequency offsets, dense Z-spectra values, populations, mutation rate, and maximum generations as the input parameters. The populations were a set of numerous non-optimal frequency offset points. The algorithm was run to output the population with the best fitness score. Each population contained chromosome values that correspond to the sparse frequency offset points. The fitness score is based on the reconstruction loss, and the algorithm operated in a closed loop, minimizing the loss each time it ran. Equation 2 represents the selection of optimal frequency offset points *F*_*optimal*_ from the set of total frequency offset points *F*_*N*_.

(3) $$F_{\text{optimal}}=\text{arg}\underset{F_{\text{selected}}\subseteq F_{N}}{\text{min}}L\left(F_{\text{selected}}\right)$$

where *L*(*F*_selected_) is the loss function. *F*_*selected*_ is the probable set of optimal frequency offsets. The objective is to minimize the loss function, i.e., to keep the Z-spectra reconstruction error as low as possible. Algorithm 1 shows the steps to select the optimal set of frequency offset points.


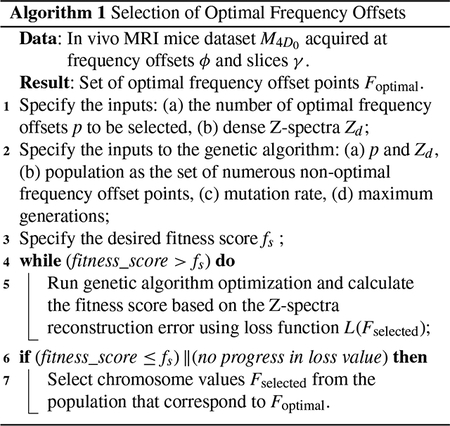


#### 2) DEEP LEARNING RECONSTRUCTION

The next step involved the reconstruction of the high-resolution Z-spectra using autoencoder and U-Net models. The models were trained with sparse Z-spectra values on optimal frequency offset points and their corresponding dense Z-spectra values. During testing, the models were provided with low-resolution Z-spectra as input, and they predicted the corresponding high-resolution Z-spectra. The study performed experiments using 10%, 20%, and 30% of the total frequency offset points. In each experiment, the model was tested with multiple low-resolution Z-spectra inputs, and the performance metrics were calculated after the prediction of high-resolution Z-spectra.

#### 3) TRAINING

**FIGURE 2.**
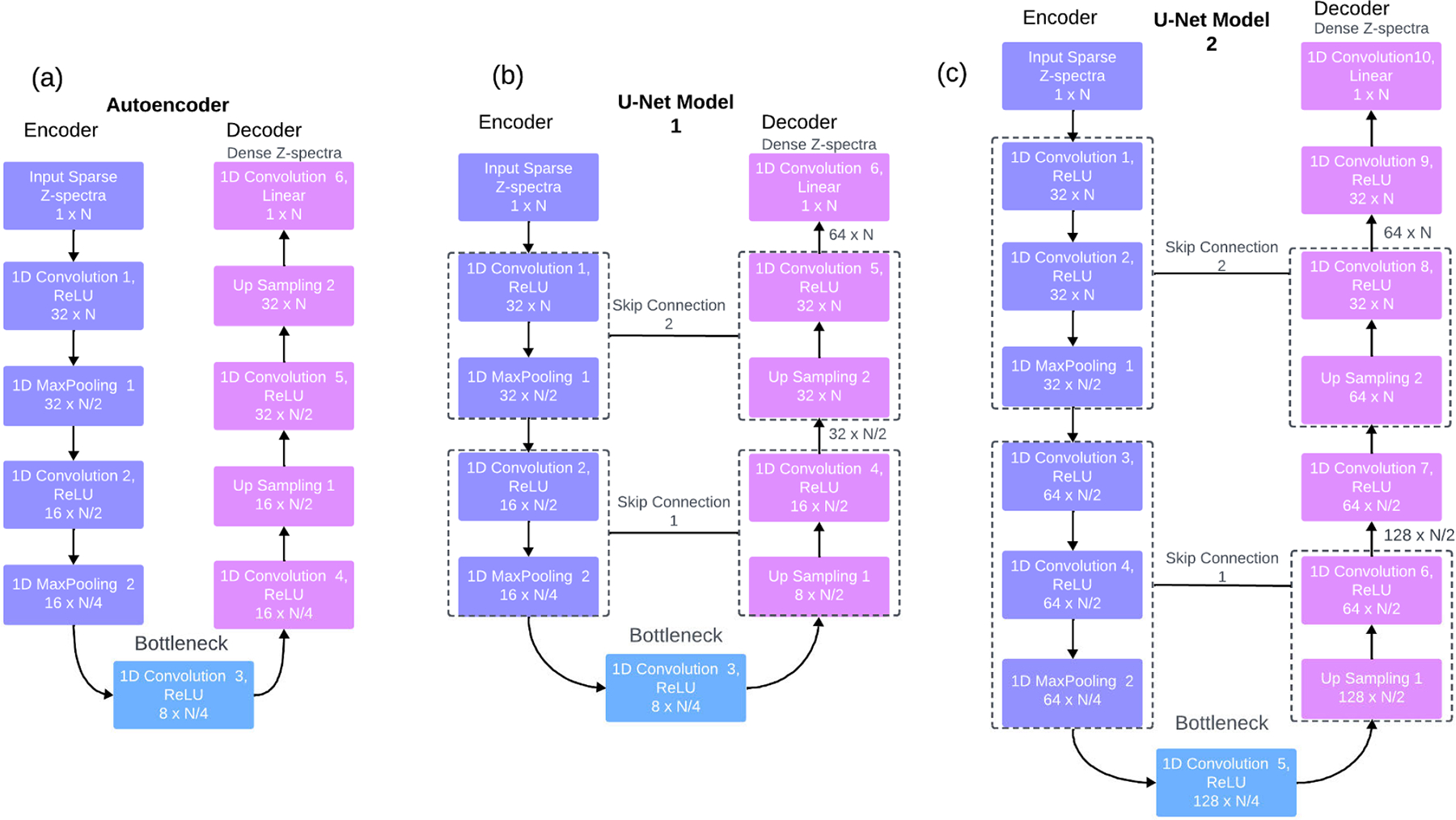
(a) The architecture of the 1D autoencoder model with 6 convolutional layers, 2 max-pooling layers, and 2 upsampling layers. The input to the model is sparse masked Z-spectra values. Input passes through the encoder, bottleneck, and decoder layers. The final output of the decoder is dense Z-spectra. In our case, N = 101 represents 101 frequency offsets. (b) The architecture of the 1D U-Net model 1. It has the same number of convolutional, max-pooling, and upsampling layers as the autoencoder model. In addition, this model has skip connections that have the potential to pass detailed features such as small shifts in Z-spectra. (c) The architecture of the 1D U-Net model 2. It has 10 convolutional, 2 max-pooling, 2 upsampling layers, and 2 skip connections. The model architecture is more complex than the autoencoder and the U-Net model 1. These three models are used to study diverse dense Z-spectra reconstructions.

We performed supervised training on the autoencoder model at first. The model architecture is shown in Figure 2(a). Likewise, we also performed supervised training on the U-Net models. In the case of the U-Net models, the concatenation layers were present to facilitate passing the feature maps from the encoder to the decoder. This mechanism likely aids in reconstructing dense Z-spectra accurately, as the skip connections enable the passing of detailed features, which may include small shifts in Z-spectra. The U-Net architectures used are shown in Figure 2(b) and Figure 2(c). The datasets were accumulated in a CSV file for training. It consisted of optimal frequency offset points and the corresponding dense Z-spectra. Autoencoder and U-Net models were trained on diverse datasets. Datasets were based on the *in vivo* datasets with and without augmentations. The training was performed using the Adam optimizer and mean absolute error loss function. A callback function reduced the learning rate if the validation loss did not improve. The minimum learning rate was set to 10^−6^. More training details are shown in Table 1.

### D. STATISTICAL ANALYSIS

First, we analyze the genetic algorithm optimization test results and later performances of the autoencoder and U-Net models. We use RMSE to observe the reconstruction accuracy of the Z-spectra. To gain further insights into the reconstruction results, we calculate MAE, which is less sensitive to outliers than the RMSE, and calculate Pearson correlation to assess how well the shape of the reconstructed Z-spectra was retained.

## IV. RESULTS

### A. OPTIMAL OFFSETS SELECTION AND Z-SPECTRA RECONSTRUCTION

#### Result 1.

**FIGURE 3.**
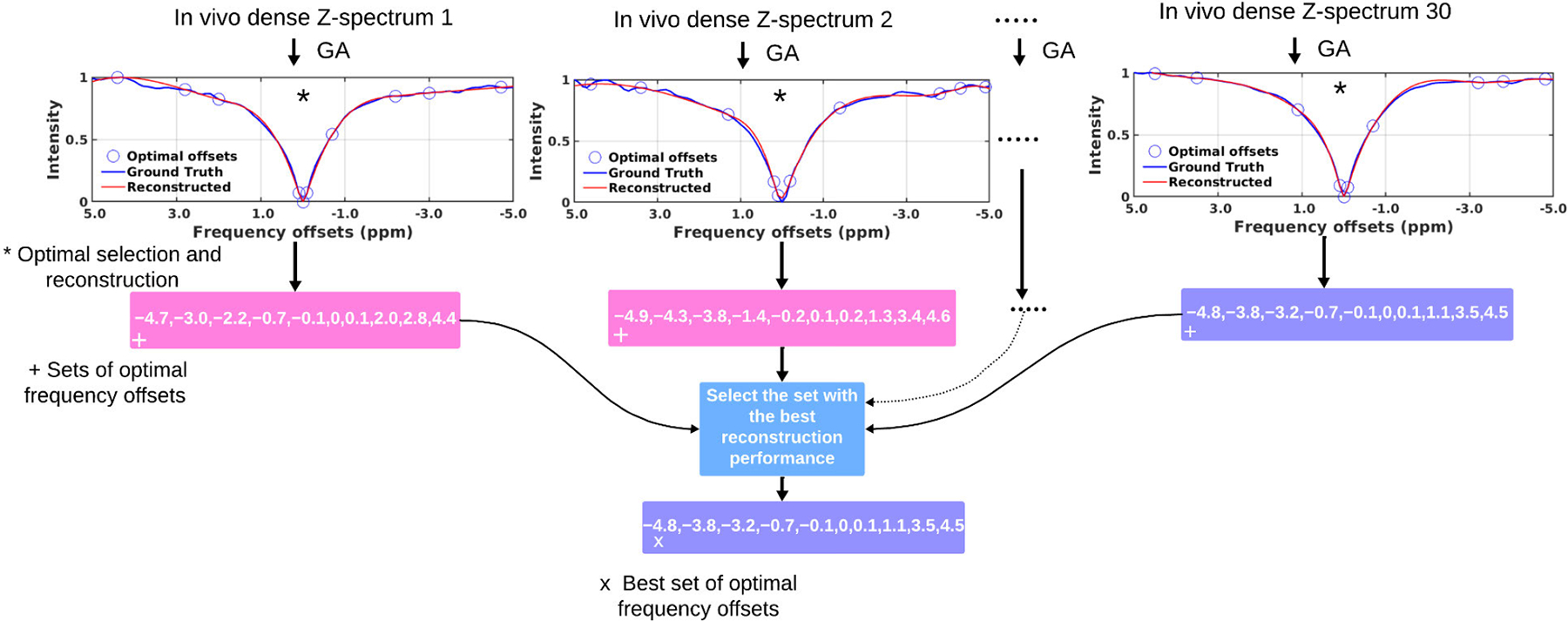
Genetic algorithm (GA) optimization was performed for *M D* 30 different in vivo dense Z-spectra, with the number of optimal offsets to be selected set to *p D* 10% (10 out of 101). Finally, the best set of optimal frequency offsets was selected based on reconstruction performance. The same method was later applied, considering 20% and 30% offsets.

The optimizations were performed on dense Z-spectra obtained from the 15 *in vivo* mice datasets, each containing 2 brain slices. 30 dense Z-spectra were obtained by calculating an average of the pixels across all the frequency offsets for each slice. The optimizations were performed individually on all these dense Z-spectra. The genetic algorithm optimization gave 30 unique sets of optimal frequency offsets for each Z-spectra. A set of frequency offsets was chosen as optimal, which, when used to reconstruct its original dense Z-spectra, resulted in a minimum reconstruction error. This approach is demonstrated in Figure 3.

Our selection of optimal frequency offset points ranged from 10% to 30% with a step of 10. The genetic algorithm selected the optimal frequency offsets for these three percentage groups. The algorithm was run to calculate the fitness score based on Z-spectra reconstruction error using the loss function. In our case, we used, spline interpolation as the initial reconstruction method, and the loss function was RMSE. The optimization terminated either when the desired loss was achieved or when there was no progress between the current and previous loss value.

#### 10%.

The optimization algorithm operated several times to select the optimal 10% frequency offset points from the 101 frequency offset points. Finally, the algorithm successfully selected the optimal frequency offsets, which, when used to reconstruct the dense Z-spectra, resulted in a minimum RMSE of 0.0127.

#### 20%.

Likewise, the genetic algorithm selected 20% of the total frequency offset points. When reconstructing the dense Z-spectra, this set of frequency offset points resulted in a minimum RMSE of 0.0070.

#### 30%.

The percentage of selection was set to 30%. When used to reconstruct the dense Z-spectra produced a minimum RMSE of 0.0036. Table 2 presents the optimal frequency offset sets corresponding to *p* = 10%, 20%, and 30%.

Dataset A: This dataset consisted of dense Z-spectra, created by calculating the average of all pixel values across the entire slice for each frequency offset. It contained 30 sets of Z-spectra for the train and 8 sets of Z-spectra for the test. In other words, 19 *in vivo* mice datasets were divided into groups of 15 and 4 for testing. Dataset A’: This dataset was based on the augmentation using additive white Gaussian noise as mentioned in Section III. Dataset B: This dataset was based on the augmentation using sub-samples of pixels across the frequency offsets for each slice as mentioned in Section III. The reconstruction models (autoencoder, U-Net model 1, and U-Net model 2) shown in Figure 2(a), Figure 2(b), Figure 2(c) were trained and tested using three different datasets (Dataset A, Dataset A’ and Dataset B).

#### Result 2.

**FIGURE 4.**
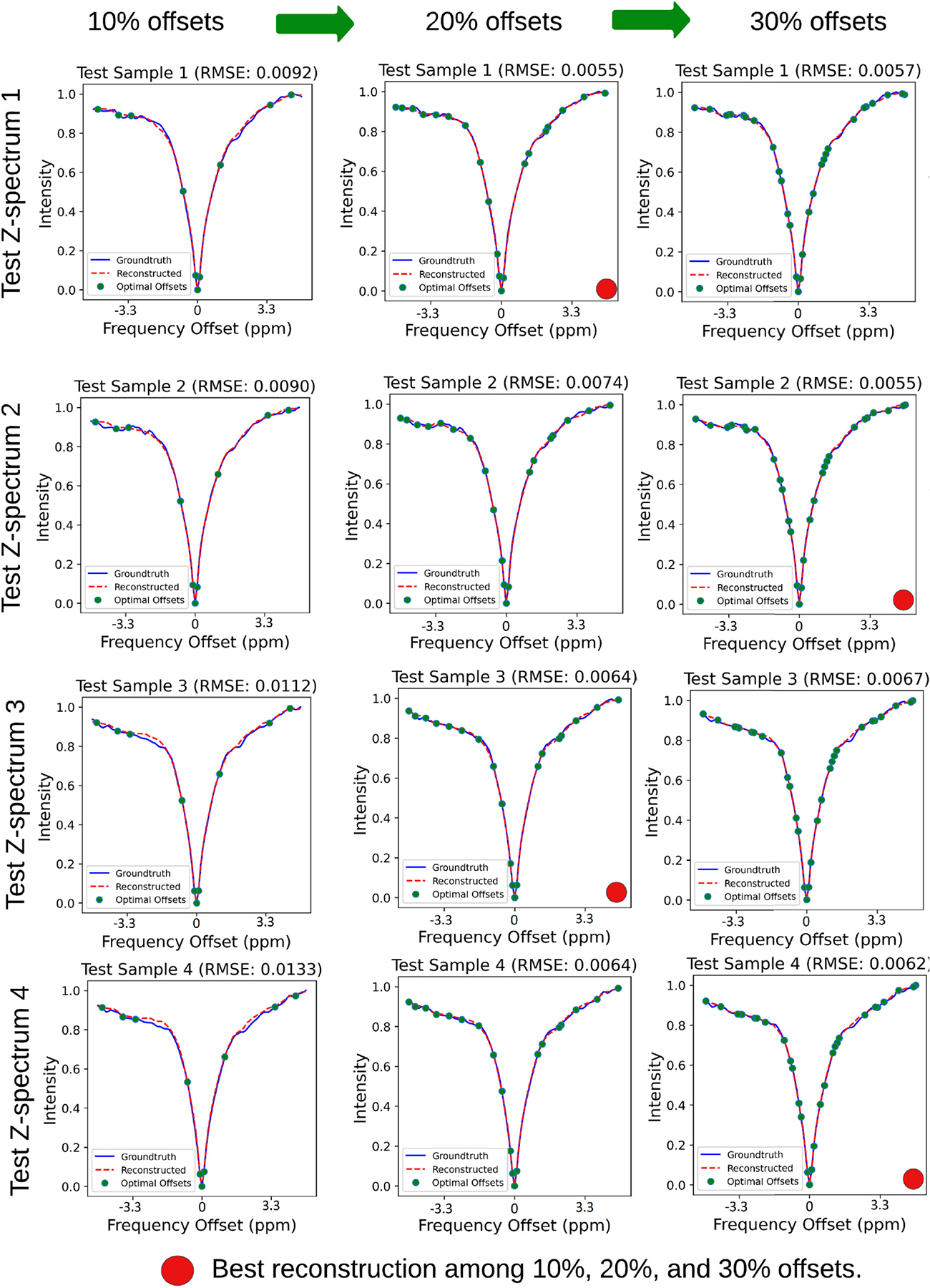
Result of the first four test Z-spectra reconstruction by autoencoder models, trained using 10%, 20%, and 30% of optimal offsets and Dataset A’, respectively. We noted the improvement in the reconstruction based on RMSE, while optimal frequency offsets were increased from 10% to 30%. However, in the case of Test Z-spectrum 1 and 3, the best performance was achieved when reconstructed with 20% optimal offsets, implying that data acquisition could be stopped in cases that meet a certain threshold value measuring the quality of reconstruction.

**FIGURE 5.**
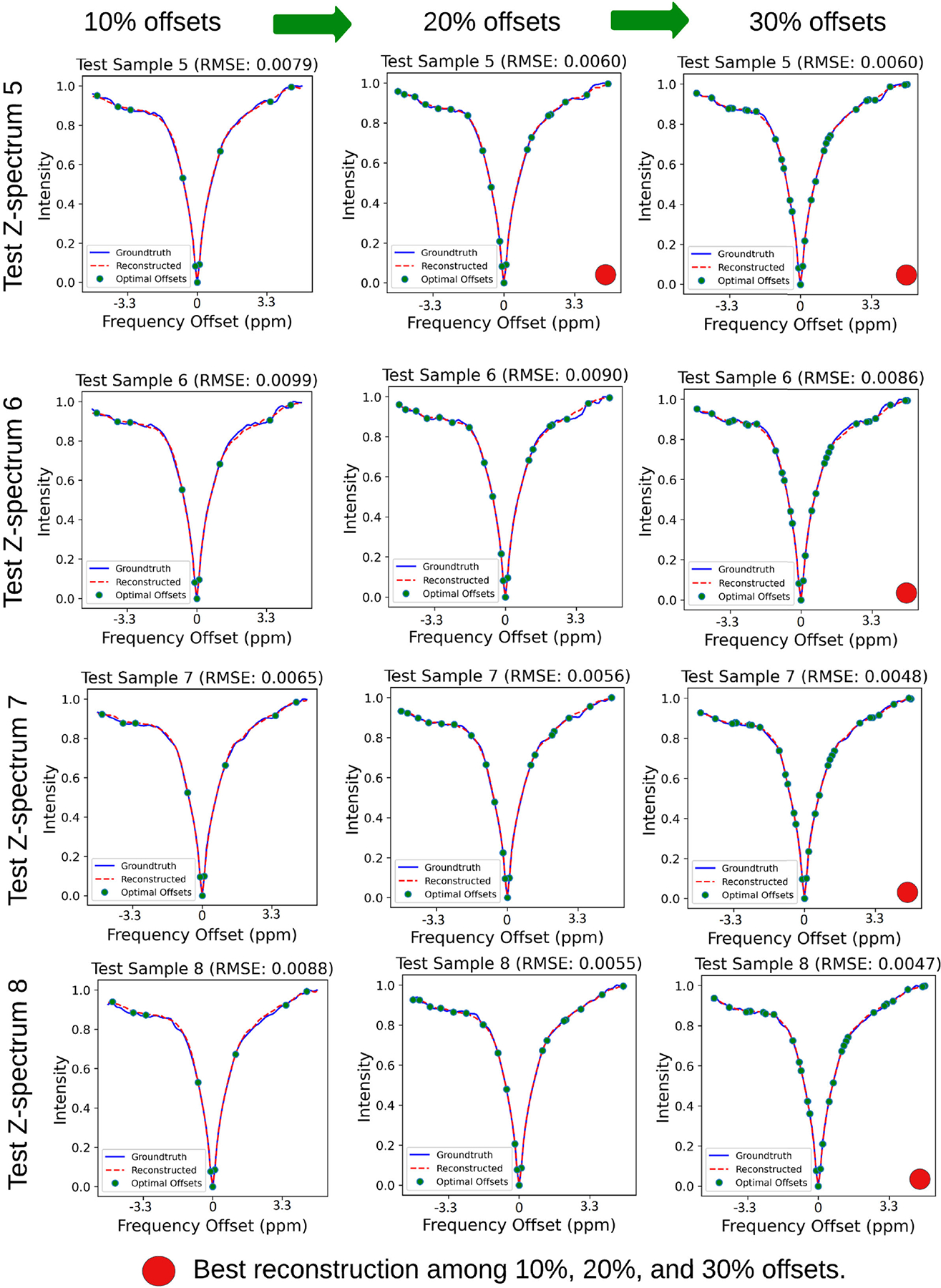
Result of the last four test Z-spectra reconstruction by autoencoder models, trained using 10%, 20%, and 30% of optimal offsets and Dataset A’, respectively. Like the first four test Z-spectra, we noted the improvement in the reconstruction based on RMSE, while optimal frequency offsets were increased from 10% to 30%. However, for Test Z-spectrum 5, the same performance was achieved with both 20% and 30% optimal offsets, suggesting that data acquisition could have been halted at 20% without the need for additional data at the 30% offsets.

The autoencoder model was trained with Dataset A’, 10 % of optimal frequency offsets, and tested with multiple Z-spectra as shown in Figure 4 and Figure 5. During the test, the low-resolution Z-spectra data with 10 frequency offsets were input to the autoencoder model to reconstruct the high-resolution Z-spectra with 101 frequency offsets. The minimum RMSE was 0.0065, the maximum RMSE was 0.0133, and the average RMSE was 0.0094 across the 8 test Z-spectra samples.

#### Result 3.

The autoencoder model was trained with Dataset A’. However, 20 % of optimal frequency offsets were selected and tested with multiple Z-spectra as shown in Figure 4 and Figure 5. During the test, the low-resolution Z-spectra data with 20% frequency offsets were input to the autoencoder model to reconstruct the high-resolution Z-spectra with 101 frequency offsets. The minimum RMSE was 0.0055, the maximum RMSE was 0.0090, and the average RMSE was 0.0064. It should be noted that the complexity of the deep learning model used for Result 3 was the same as for Result 2, but more optimal frequency offsets were involved. This resulted in better reconstruction performance. We pointed out that the performance improved by 15.38% for the minimum RMSE, by 32.33% for the maximum RMSE, and by 31.91% for the average RMSE compared to Result 2.

#### Result 4.

The autoencoder model was trained with Dataset A’. However, 30 % of optimal frequency offsets were selected, and tested with multiple Z-spectra as shown in Figure 4 and Figure 5. During the test, the low-resolution Z-spectra data with 30 frequency offsets were input to the autoencoder model to reconstruct the high-resolution Z-spectra with 101 frequency offsets. The minimum RMSE was 0.0047, the maximum RMSE was 0.0086, and the average RMSE was 0.0059. Once again, the same autoencoder model was used as in Results 2 and 3, and the optimal frequency offsets were increased from 20% to 30%. We noted that the performance improved by 14.54% for the minimum RMSE, by 4.44% for the maximum RMSE, and by 7.81% for the average RMSE compared to Result 3. However, when optimal offsets increased from 20% to 30%, some reconstruction performances either degraded or remained identical. These results implied that data acquisition could be stopped in cases that meet a certain threshold value measuring the quality of reconstruction. This would ultimately help optimize the acquisition time for CEST MRI by avoiding data collection at those offsets that do not further aid in improving the quality of reconstruction.

### B. COMPARISON OF FREQUENCY OFFSETS SELECTION APPROACHES

In this comparative study, we considered the 11 frequency offsets selected by Xiao et al. in their research work [19]. Moreover, 10 frequency offsets were selected by a pseudo-random number generator (PRNG), and 10 optimal frequency offsets were selected by GA. We utilized four test datasets, each containing two brain slices, which gave a total of 8 Z-spectra by averaging the pixels across offsets. We tested three selection approaches with those 8 Z-spectra. We used the pre-trained U-Net model 2 to reconstruct the dense Z-spectra and compared the results based on reconstruction performance measured by RMSE.

**FIGURE 6.**
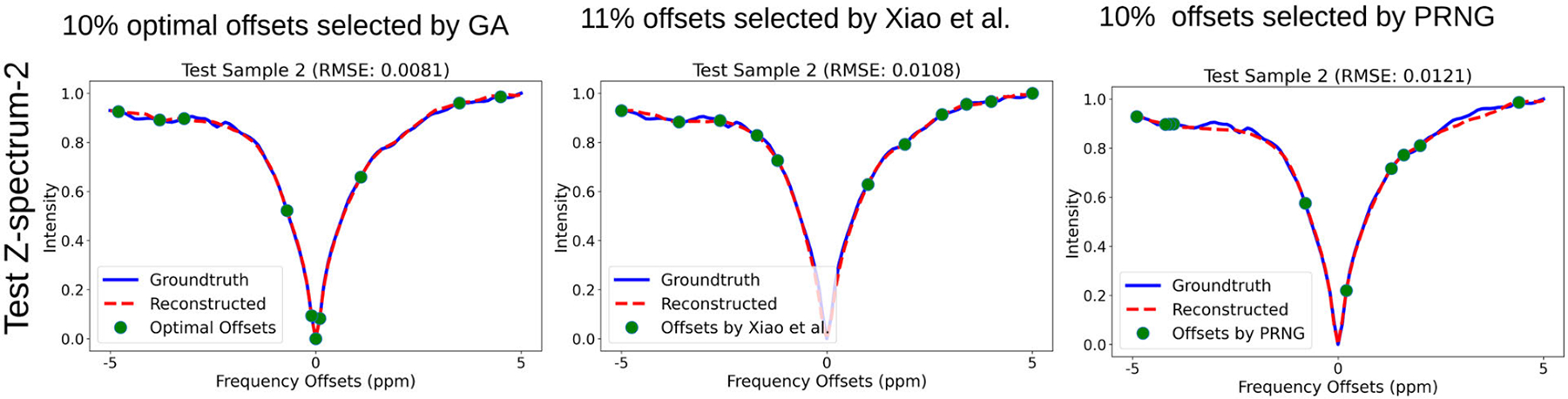
Reconstruction performance of the test Z-spectrum 2 using three frequency offset selection approaches. The GA approach, selecting 10% of optimal offsets, achieved an RMSE of 0.0081, which is better than the 11% offsets selected by Xiao et al., with an RMSE of 0.0108. The PRNG approach, selecting 10% of offsets, had an RMSE of 0.0121, which was higher than that of the other two approaches.

From Table 3, we see that only 10% of the optimal offsets were used in the case of the GA selection approach, yet we obtained performance comparable to that of the offsets selected by Xiao et al. The average RMSE for the three approaches (Xiao et al., GA, and PRNG) was 0.0086, 0.0091, and 0.0106, respectively. In the case of PRNG, it also produced good performance, but the average RMSE was lower than that of the other two approaches. Figure 6 shows the reconstruction performance of test Z-spectrum 2 using three frequency offset selection approaches, while further details of the other tests can be found in Table 3.

### C. STATISTICAL RESULTS AND ANALYSIS

#### Result 5.

**FIGURE 7.**
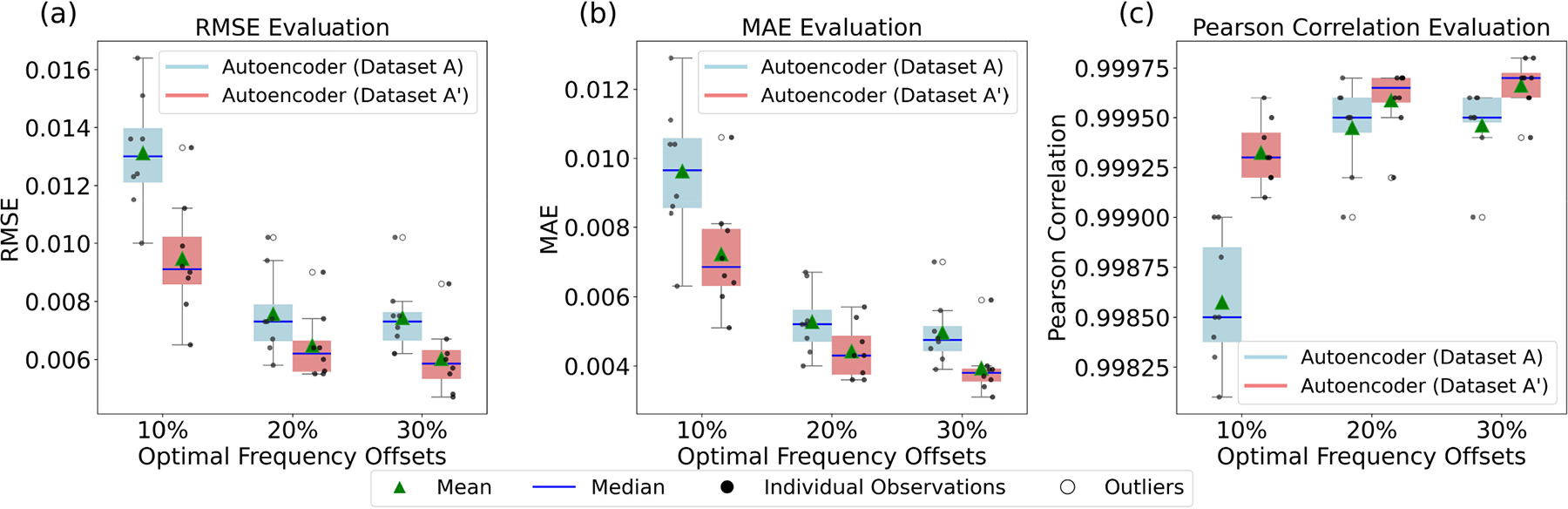
Plots compare the reconstruction performance of autoencoder models on three metrics: root mean square (RMSE), mean absolute error (MAE), and Pearson correlation. (a) RMSE across different optimal frequency offsets (10%, 20%, 30%) for autoencoder models trained with Dataset A and Dataset A’. (b), (c) MAE and Pearson Correlation across the same offsets. We observed that models reconstructed Z-spectra with very low errors across various optimal frequency offsets, and performance improved when optimal frequency offsets were increased. Moreover, it was inferred that the autoencoder model trained with augmented data showed better performance.

The in-depth analysis of the dense Z-spectra reconstruction results considered the calculation of three metrics: RMSE, MAE, and Pearson correlation. We considered the percentage of selected optimal frequency offset points (10% to 30%), and the deep learning models (autoencoders) trained on Dataset A and Dataset A’. Figure 7 presents the box plots that compare the reconstruction performances of models across different optimal frequency offsets.

#### Analysis of Figure 7.

In Figure 7(a), RMSE values are seen as relatively higher compared to MAE values in Figure 7(b). This was because RMSE was more sensitive to large reconstruction errors than MAE. However, MAE gave an idea of the average Z-spectra reconstruction performance across various optimal frequency offsets. In the case of, 7(c), Pearson correlation showed how well the model predicted the Z-spectra in terms of shape rather than focusing on exact magnitudes of errors. Most of the Pearson correlations for Z-spectra reconstructions were close to 1. This meant that the shape of Z-spectra was retained very well.

#### Result 6, 7 and 8.

**FIGURE 8.**
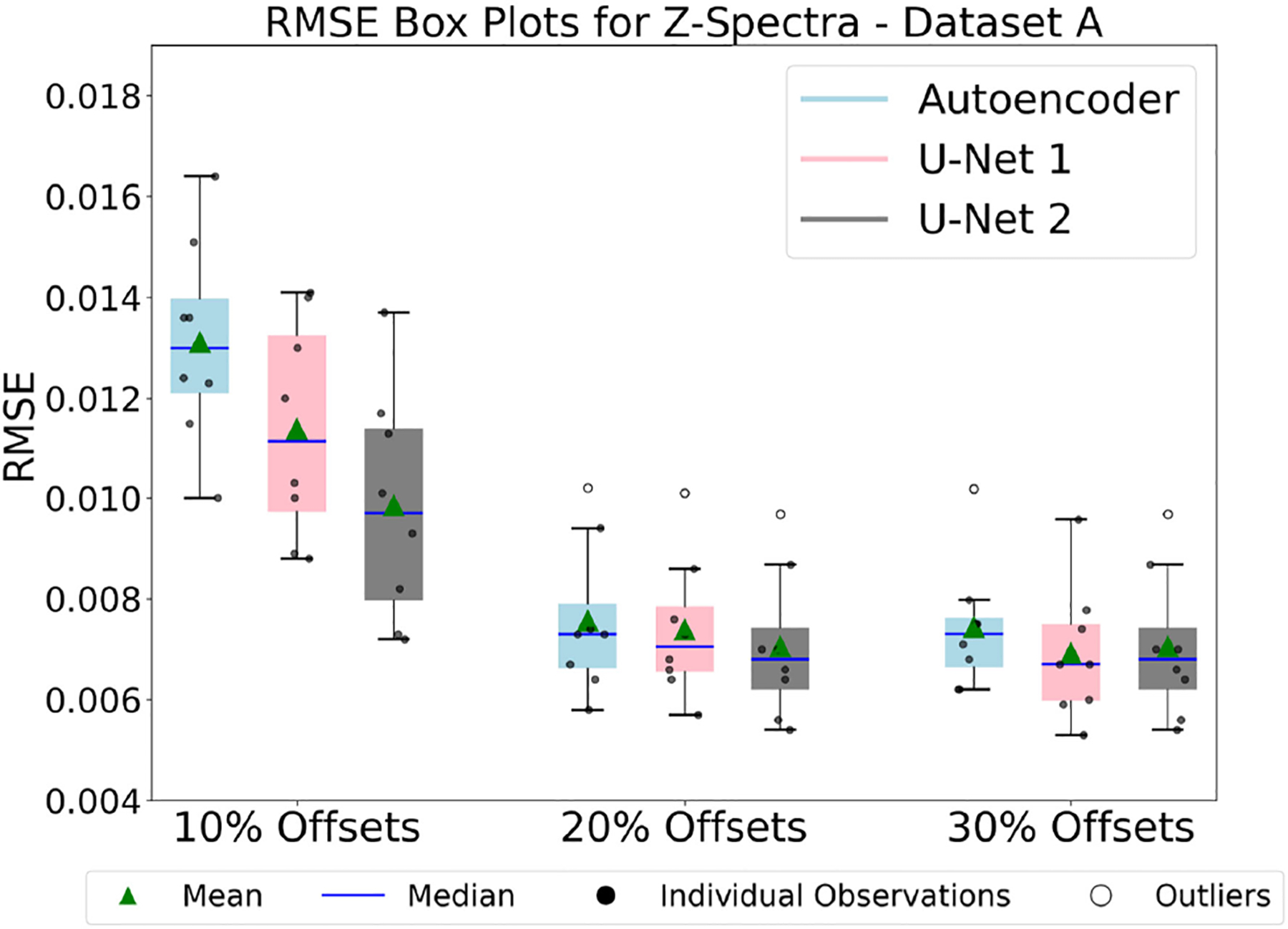
The figure compares the performances of three models: Autoencoder, U-Net model 1, and U-Net model 2, by showing the box plot of RMSE for reconstructed dense Z-spectra (using 10%, 20%, and 30% optimal frequency offsets) from models trained on Dataset A. The U-Net model 2 showed the best performance, but the performance was comparable to the autoencoder and U-Net model 1.

**FIGURE 9.**
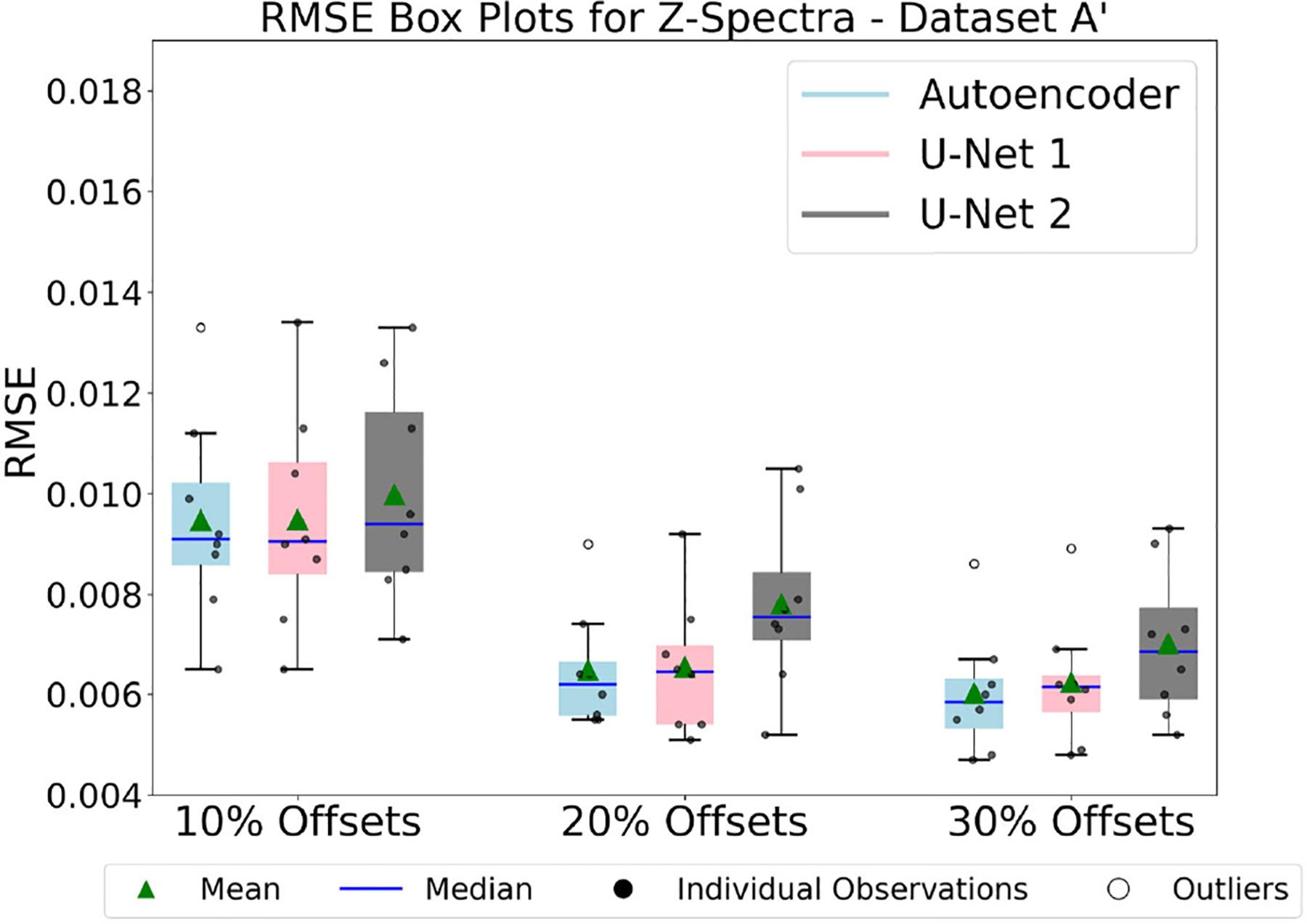
The figure compares the performances of three models: Autoencoder, U-Net model 1, and U-Net model 2, by showing the box plot of RMSE for reconstructed dense Z-spectra (using 10%, 20%, and 30% optimal frequency offsets) from models trained on Dataset A’. It was inferred that data augmentation mostly benefited the performance of the autoencoder model, particularly for the 10% offsets experiments.

**FIGURE 10.**
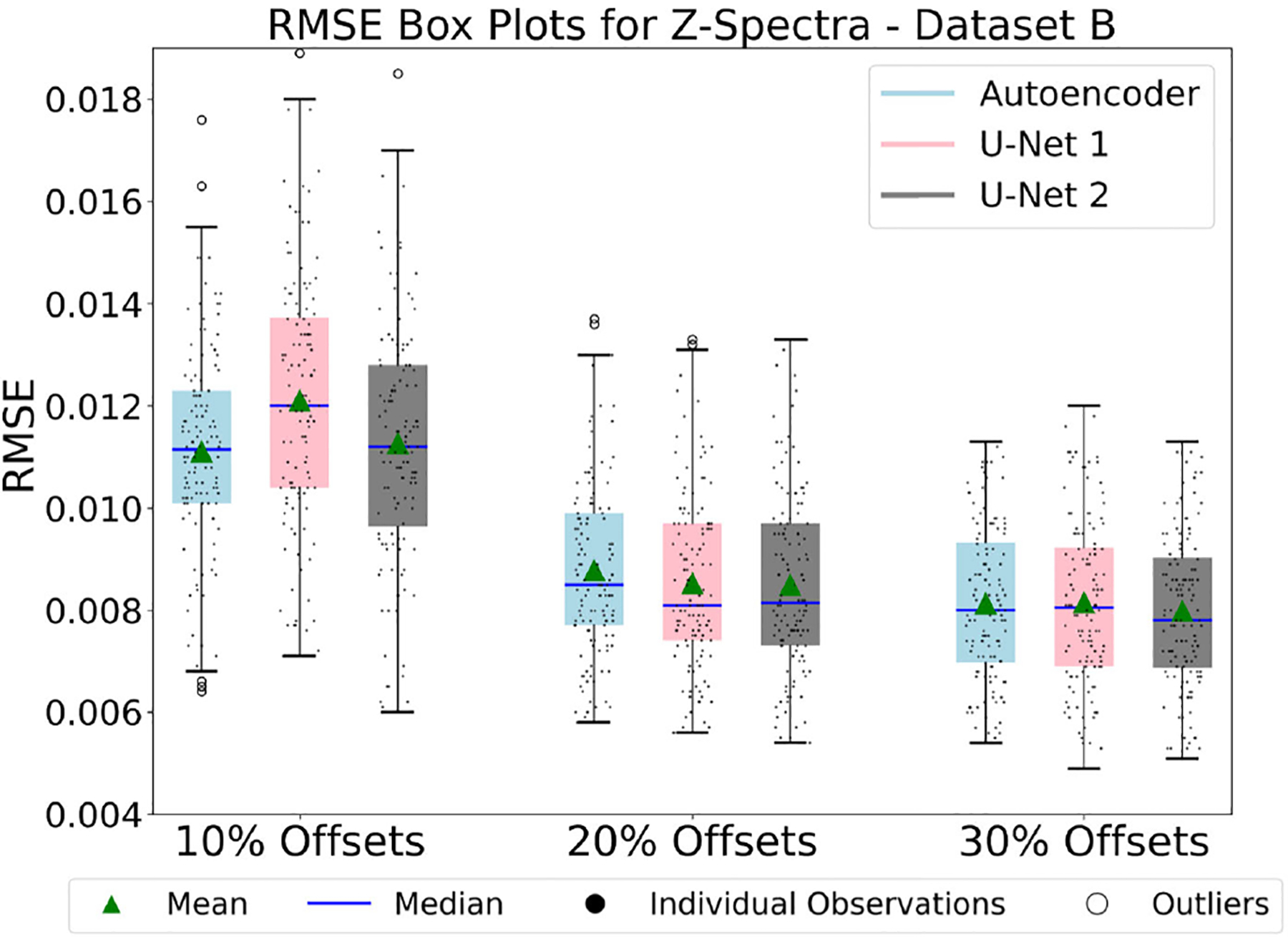
Figure compares the performances of three models (Autoencoder, U-Net model 1, and U-Net model 2) by presenting box plots of RMSE for reconstructed dense Z-spectra (using 10%, 20%, and 30% optimal frequency offsets) from models trained on Dataset B. The U-Net model 2 demonstrated the best performance and was able to effectively adapt to diverse Z-spectra reconstructions, particularly when the percentage of frequency offsets was 30%.

The plots in Figure 8, Figure 9, and Figure 10 compare the RMSE of Z-spectra reconstructions produced by various models trained on Dataset A, Dataset A’ and Dataset B. Specifically, these figures compare the RMSE of Z-spectra reconstructions produced by autoencoder and U-Net models with 10%, 20%, and 30% of frequency offsets, respectively.

#### Result 6 and analysis of Figure 8.

The results correspond to 9 different training configurations on Dataset A. Each model was trained using p = 10%, 20%, and 30% of the optimal frequency offsets and tested across three different models. As shown in Figure 8, the RMSE results for these 9 configurations are presented. For all configurations, the performance improved as the model complexity and percentage of optimal frequency offsets were increased. However, the models tended to achieve comparable performances when more optimal frequency offsets were used during reconstructions. As an example, when 30% of optimal frequency offsets were considered, the U-Net model 2 showed the best performance, but the performance was comparable to the autoencoder and U-Net model 1.

#### Result 7 and Analysis of Figure 9.

It shows the results of 9 different combinations like Figure 8, and therefore, 9 different trainings on Dataset A’ were performed to achieve these results. The performance improved when the percentage of optimal frequency offsets was increased. The autoencoder model performed best across all three sets of optimal frequency offsets. Nonetheless, the reconstruction performances of U-Net models were still satisfactory and comparable to the autoencoder model. It should be noted that all 9 models were tested with the same 8 *in vivo* Z-spectra test samples to have a better analysis of the reconstruction performances. Unlike the results involving 10% offsets shown in Figure 8, the performance of the autoencoder was comparable to that of U-Net 1 and U-Net 2, as shown in Figure 9.

This finding indicates that, in the case of 10% offsets, the performance improved specifically for the autoencoder models upon augmenting the dataset, whereas the performance of the U-Net 1 and U-Net 2 models remained similar. This can be observed by analyzing Figure 8 and Figure 9. Results indicate that trade-offs exist among datasets, model architecture, and the percentage of optimal frequency offsets.

From these results, we concluded that the training and testing performance improved upon augmenting Dataset A for lower percentages of frequency offsets, such as 10%. It was the autoencoder model that showed the best performance. To get further insights, the comparison of the performance of the autoencoder model before and after the augmentation was shown in Figure 7 and discussed in Result 5.

#### Result 8 and analysis of Figure 10 and 11.

It shows 9 training results on Dataset B. Dataset B was obtained through augmentation using subsamples as mentioned in Section III. There were 128 diverse *in vivo* dense Z-spectra samples to test the reconstruction performances of the models. Compared to previous results on Dataset A and Dataset A’, this result provided insights into the relationship between the complexity of the model’s architecture, the number of optimal frequency offsets, and the nature of the datasets.

As shown in Figure 10, the reconstruction performance improved when the percentage of optimal frequency offsets was increased. Moreover, the reconstruction performance improved in all cases as the model complexity increased, except for the U-Net model 1, where performance degraded slightly in the case of 10% of the optimal frequency offsets. Compared to the results obtained for Dataset A and Dataset A’ in Figure 8 and Figure 9, the reconstruction performance on Dataset B based on RMSE was relatively lower. It should be noted that the Z-spectra in Dataset B were more diverse compared to the Z-spectra in Dataset A and Dataset A’ because these datasets were formed from the sub-samples started with 800 pixels and increased up to 1550 pixels in increments of 50. Moreover, models trained with Dataset B were tested with 128 diverse Z-spectra, compared to 8 Z-spectra for Dataset A and Dataset A’. This likely explains the relatively lower performance of the models on Dataset B.

**FIGURE 11.**
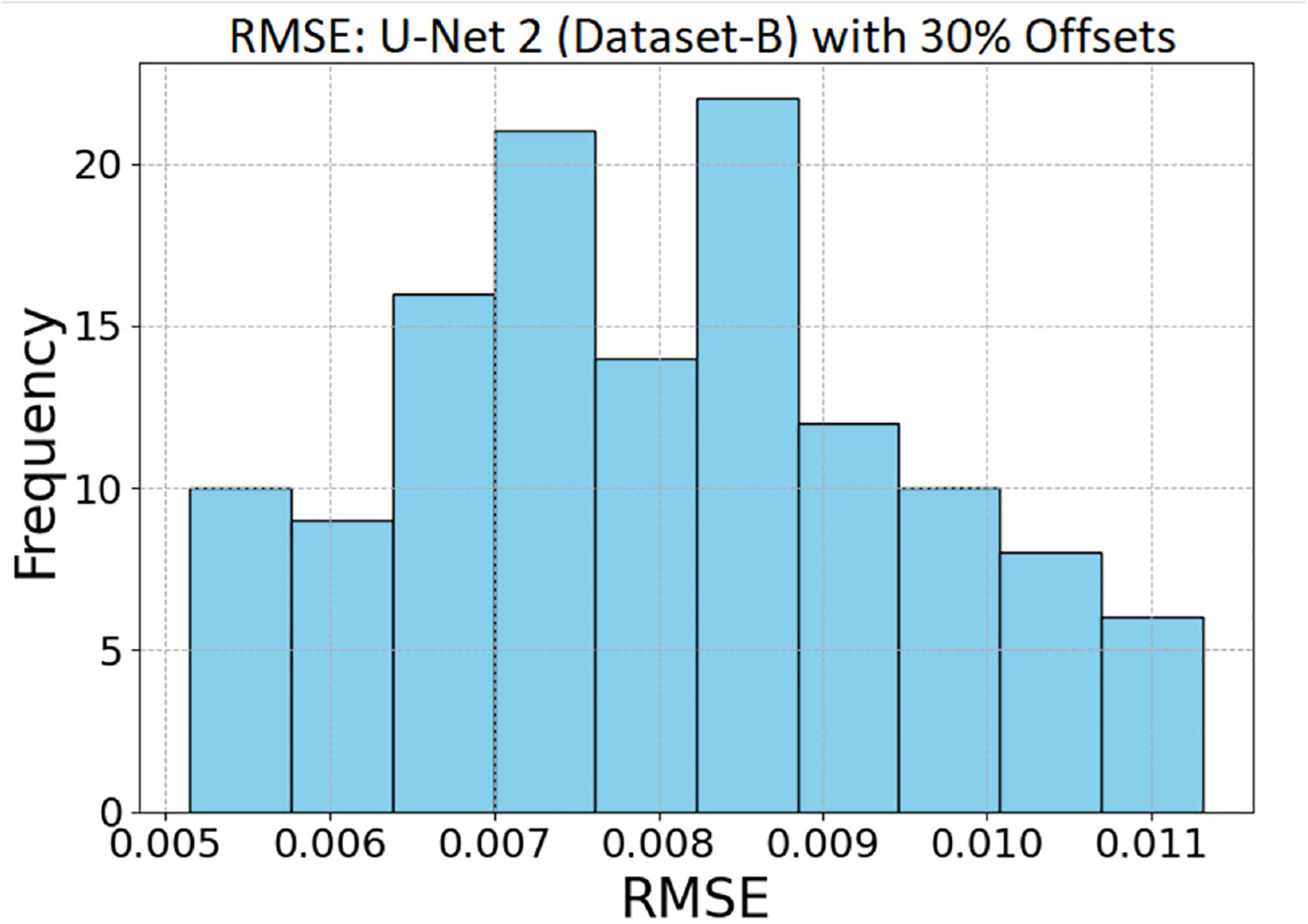
Distribution of RMSE values for 128 in vivo Z-spectra reconstructed using the U-Net 2 model with 30% of the optimal frequency offsets. U-Net model 2 showed a tighter range of RMSE values. This indicated that the model performed better in the 30% frequency offset experiments.

Figure 10 shows that all three models (autoencoder, U-Net 1, and U-Net 2) with 30% of optimal frequency offsets demonstrated impressive performance. In comparison, the U-Net model 2 showed slightly better performance. To gain further insights into this performance, we plotted the distribution of the RMSE. Figure 11 illustrates the distribution of RMSE values, with the x-axis representing RMSE and the y-axis representing the frequency of occurrences, obtained from reconstructing 128 dense Z-spectra using the U-Net model 2. The minimum RMSE was 0.0051, the maximum RMSE was 0.0113, and the average RMSE was 0.00798 across the 128 *in vivo* reconstructed Z-spectra samples. U-Net model 2 exhibited a narrower range of RMSE values. This suggested that the model performed better in the 30% frequency offset experiments.

### D. MAGNETIZATION TRANSFER RATIO MAPS

**FIGURE 12.**
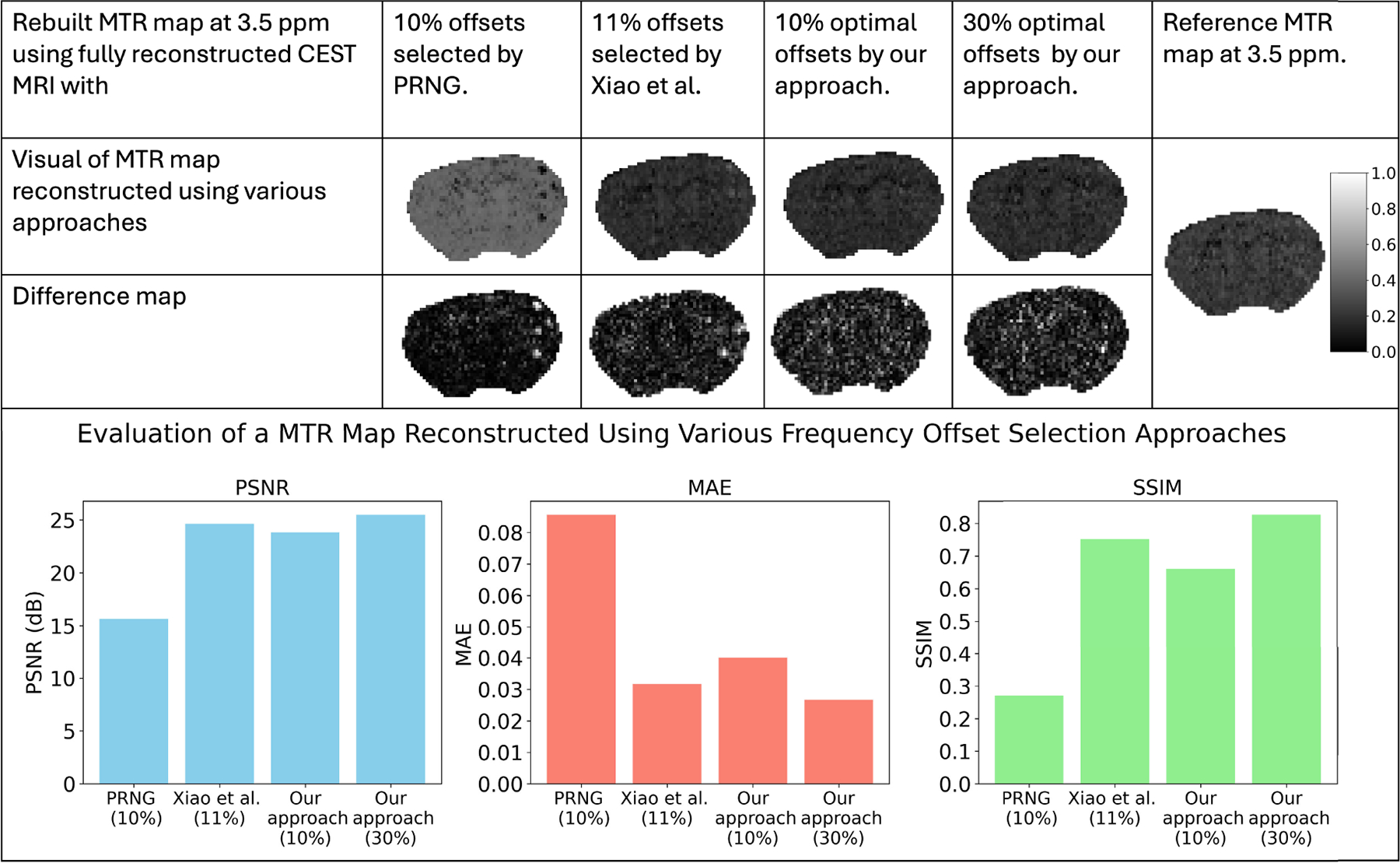
Reconstruction of the MTR maps at 3.5 ppm using fully reconstructed CEST MRI from sparse data, based on various frequency offset selection approaches. The maps were obtained from the fully reconstructed data, which were generated using 10% offsets selected by PRNG, 11% offsets selected by Xiao et al., and the optimal 10% and 30% offsets selected by our approach. In addition, the difference map, reference map, and evaluation based on PNSR, MAE, and SSIM are presented. It was inferred that our approach performs comparably to the empirical approach (Xiao et al.) and outperforms PRNG. Further, the reconstruction quality improved notably as we increased to the optimal 30% offsets.

We reconstructed magnetization transfer ratio (MTR) maps at 3.5 ppm using fully reconstructed CEST MRI data derived from sparse data. U-Net Model 2 was used to reconstruct the full CEST MRI data from sparse data. Those sparse data represented fully sampled CEST MRI collected at limited frequency offsets. Various sets of sparse frequency offsets were selected using different approaches. Those approaches were 10% offsets selected by PRNG, 11% offsets selected by Xiao et al. [19], and the optimal 10% and 30% offsets selected by our approach. We compared the rebuilt MTR maps based on these frequency selection approaches. The peak signal-to-noise ratio (PSNR), the mean absolute error (MAE), and the structural similarity index measure (SSIM) were calculated for each reconstructed MTR map and compared using a bar graph, as shown in Figure 12. From the bar graph, it was observed that our approach, which considered 10% optimal offsets, performed comparably to the empirical approach of Xiao et al., which selected 11% offsets. In addition, our approach outperformed the PRNG approach that selected random offsets 10%. Please note that our approach, different from Xiao et al.’s approach, offers a systematic methodology as an optimization framework to seek and identify efficient frequency offsets. Moreover, we observed that the reconstruction quality, measured by PSNR, MAE, and SSIM, improved notably as the optimal offset percentage increased to 30%.

## V. DISCUSSION

The objective of this research was to develop a systematic method to improve the efficiency of CEST MRI by reducing data acquisition time. This was achieved by optimizing the selection of frequency offset points, followed by deep learning Z-spectra reconstruction. Results show that the optimization algorithm, such as the genetic algorithm, could select optimal frequency offset points down to 10% of the total frequency offsets. Furthermore, the Z-spectra reconstruction using those optimal frequency offset points with autoencoder and U-Net models trained on various datasets resulted in a lower reconstruction error. This supports our hypothesis that the two-step procedure involving optimization followed by deep learning reconstruction improves the efficiency of CEST MRI data acquisition.

While previous studies by Cheema et al. show that frequency offsets can be optimized to as low as 28.30% (15 out of 53) using their approach of Fisher information gain analysis [13], our findings indicate that it can be reduced up to 10%. Further, Chen et al. selected 25.54% (13 out of 51) frequency offsets to reconstruct rat brain dense Z-spectra using their proposed approach of Z-spectral compressed sensing to obtain the best results with a minimum RMSE of 0.0049 [14]. However, our findings indicate we can achieve RMSE as low as 0.0065 using only 10% of optimal frequency offsets to reconstruct mice brains Z-spectra.

These findings have significant implications for the development of an efficient CEST MRI method, particularly in reducing data acquisition time, thus expanding the applications of the method. Consequently, faster CEST MRI acquisition would advance *in vivo* molecular bioimaging for basic science and clinical research as well as for clinical theranostic applications.

The limitation of the study was that limited *in vivo* CEST MRI data were used as scan data were expensive to acquire. However, augmentation was performed to enhance the training performance of deep learning models. The models were tested using both augmented and original *in vivo* mice brain Z-spectra, and the results were compared. For optimization, spline interpolation was utilized to reconstruct the dense Z-spectra and assess the reconstruction error. Our findings suggested that the deep learning reconstruction later significantly reduced the reconstruction error. Such observation was potentially due to optimal frequency offsets selection by the genetic algorithm.

Future studies should consider more *in vivo* CEST MRI datasets, including both healthy and disease groups. This may require a more robust optimization technique to select optimal frequency offsets and complex deep-learning reconstruction models for adapting to diverse Z-spectra.

In summary, this study concludes that the optimal selection of frequency offsets followed by deep learning reconstruction is a promising approach for addressing limitations associated with extended time requirement for CEST MRI data acquisition, laying the groundwork for efficient *in vivo* molecular bioimaging.

## VI. CONCLUSION

The research aimed to investigate a systematic method to optimize CEST MRI data acquisition, providing insights into the selection of optimal frequency offsets to reconstruct accurate dense Z-spectra. Our findings indicate that the percentage of optimal selection of frequency offsets can reach a lower value than the current state-of-the-art considers, and can achieve acceptable reconstruction accuracy. This study paves a strong foundation for the development of a more efficient CEST MRI method, expanding its applications in both pre-clinical and clinical research. This study offers meaningful insights but is limited by the use of a small amount of *in vivo* CEST MRI data due to the high cost of acquisition. However, an effort was made to augment the data. Future research would be built on this platform, and a larger sample size would be utilized for adapting to diverse Z-spectra. Such an approach would help to develop a more generalizable system to optimize the selection of frequency offsets and reconstruct high-quality Z-spectra, ultimately enhancing basic science and clinical studies and related applications.

## Figures and Tables

**TABLE 1.** Training details for all deep learning models used in the study.

Description	Value
**Training Data**	Optimal frequency offsets (10%, 20% and 30%) and dense Z-spectra
**Epochs**	Between 250 and 500 for different models
**Batch Size**	Between 5 and 25 for different models
**Validation Split**	0.2
**Compute Resources**	JupyterLab version 4.0, facilitated by the Swan cluster, operated by the Holland Computing Center (HCC) (requested 8 cores, 32 GB RAM)

**TABLE 2.** Optimal frequency offsets selected by the optimization algorithm.

*p*%	Optimal Frequency Offsets (ppm)
10%	−4.8, −3.8, −3.2, −0.7, −0.1, 0.0, 0.1, 1.1, 3.5,4.5
20%	−5.0, −4.7, −4.2, −3.7, −3.1, −2.5, −1.7, −1.0, −0.6, −0.2, −0.1, 0.0, 0.1, 1.1, 1.3, 2.1, 2.2, 2.9, 3.9,4.9
30%	−4.9, −4.2, −3.4, −3.3, −3.2, −2.6, −2.5, −2.1, −1.2, −0.9, −0.8, −0.5, −0.4, −0.1,0.0, 0.1,0.2, 0.5, 0.7,1.1, 1.2, 1.3,1.4, 2.6, 3.1, 3.2, 3.5,4.2, 4.9, 5.0

**TABLE 3.** RMSE observed during the reconstruction of various Z-spectra using different offset selection approaches: 10% optimal offsets selected by GA, 11% offsets selected by Xiao et al., and 10% offsets selected by PRNG.

Test Z-spectrum	GA	Xiao et al.	PRNG
**Test Z-spectrum 1**	0.0085	0.0076	0.0112
**Test Z-spectrum 2**	0.0081	0.0108	0.0121
**Test Z-spectrum 3**	0.0105	0.0090	0.0114
**Test Z-spectrum 4**	0.0125	0.0076	0.0159
**Test Z-spectrum 5**	0.0077	0.0085	0.0082
**Test Z-spectrum 6**	0.0097	0.0093	0.0127
**Test Z-spectrum 7**	0.0060	0.0089	0.0058
**Test Z-spectrum 8**	0.0100	0.0077	0.0069
**RMSE Analysis**			
**Minimum RMSE**	0.0060	0.0076	0.0058
**Maximum RMSE**	0.0125	0.0108	0.0159
**Average RMSE**	0.0091	0.0086	0.0105
